# Determination of phage load and administration time in simulated occurrences of antibacterial treatments

**DOI:** 10.3389/fmed.2022.1040457

**Published:** 2022-10-28

**Authors:** Steffen Plunder, Markus Burkard, Ulrich M. Lauer, Sascha Venturelli, Luigi Marongiu

**Affiliations:** ^1^Department of Mathematics, University of Vienna, Vienna, Austria; ^2^Department of Nutritional Biochemistry, University of Hohenheim, Stuttgart, Germany; ^3^Department of Internal Medicine VIII, University Hospital Tübingen, Tübingen, Germany; ^4^Department of Vegetative and Clinical Physiology, Institute of Physiology, University Hospital Tübingen, Tübingen, Germany

**Keywords:** microbial ecology models, phage therapy, machine learning, Pareto optimization, antibacterial treatment

## Abstract

The use of phages as antibacterials is becoming more and more common in Western countries. However, a successful phage-derived antibacterial treatment needs to account for additional features such as the loss of infective virions and the multiplication of the hosts. The parameters critical inoculation size (*V*_*F*_) and failure threshold time (*T*_*F*_) have been introduced to assure that the viral dose (*V*_ϕ_) and administration time (*T*_ϕ_) would lead to the extinction of the targeted bacteria. The problem with the definition of *V*_*F*_ and *T*_*F*_ is that they are non-linear equations with two unknowns; thus, obtaining their explicit values is cumbersome and not unique. The current study used machine learning to determine *V*_*F*_ and *T*_*F*_ for an effective antibacterial treatment. Within these ranges, a Pareto optimal solution of a multi-criterial optimization problem (MCOP) provided a pair of *V*_ϕ_ and *T*_ϕ_ to facilitate the user’s work. The algorithm was tested on a series of *in silico* microbial consortia that described the outgrowth of a species at high cell density by another species initially present at low concentration. The results demonstrated that the MCOP-derived pairs of *V*_ϕ_ and *T*_ϕ_ could effectively wipe out the bacterial target within the context of the simulation. The present study also introduced the concept of mediated phage therapy, where targeting booster bacteria might decrease the virulence of a pathogen immune to phagial infection and highlighted the importance of microbial competition in attaining a successful antibacterial treatment. In summary, the present work developed a novel method for investigating phage/bacteria interactions that can help increase the effectiveness of the application of phages as antibacterials and ease the work of microbiologists.

## Introduction

First employed in the medical field about a century ago, bacteriophages (phages) are currently experiencing a renewed clinical and veterinary interest particularly for their potential to contain antibiotic-resistant bacteria (1, 2). Phages are employed, albeit still in an experimental way, to treat clinical bacterial infections (3) including those due to antibiotic resistant species (4, 5), resolve caries (6, 7), preserve food, and decontaminate livestock (8). Although phages will likely be used in conjunction with antibiotics, at least in clinical settings (9, 10), their broad range of applications necessitates a deep understanding of their behavior to predict the efficacy of the treatment. Because phages are not static entities but rather replicate in proportion to their hosts’ density, failing to account for this characteristic may result in therapeutic failure (11). To establish a self-sustaining infectious chain, there is the need for a minimum concentration of hosts for the phages known as “proliferation threshold” (12, 13):


(1) $$X_{P}\approx \frac{\lambda \,\left(\eta -\mu \right)}{\delta \,\beta \,\eta }$$

where μ is the growth rate of the bacterial host, and the other parameters are the life-history tracts of the phages (λ = decay rate; η = reciprocal of the latency time τ; δ = adsorption rate; β = burst size). *X*_*P*_ is reached at a time known as “proliferation onset time”:


(2) $$T_{P}\approx \frac{1}{\mu }ln\left(\frac{\lambda (\eta -\mu )}{\delta \eta \beta N}\right)$$

where *N* is the total bacterial population. These parameters depend on each pair of bacteria and phages and provide a guidance on the possible outcome of the phage application. Consequently, if phages are given before *T*_*P*_, they will not replicate successfully. However, if the viral load administered (*V*_ϕ_) is high enough, phages will massively lyse their hosts even in the absence of replication, and the treatment (known as “passive”) will resemble antibiotic features where the drug does not amplify once administered. Conversely, if the administration time (*T*_ϕ_) is occurring after *T*_*P*_, the phage-derived antibacterial treatment is defined as “active” because the virus will actively replicate establishing a self-sustained infectious cycle.

The parameter “critical inoculation size” (*V*_*F*_) was introduced to provide a guide to the minimum amount of phages that *V*_ϕ_ should be administered to achieve an effective therapy (12). The critical inoculation size is defined as:


(3) $$V_{F}=\varepsilon \,e\,x\,p\,\left(\omega \,\left(T_{P}-T_{\phi }\right)+\frac{\omega }{\mu }\,e^{-\mu \,\left(T_{P}-T_{\phi }\right)}-1\right)$$

where ε is the dilution factor to obtain one phage in the system and ω is the decay or wash-out of the microbes. Similarly, the “failure threshold time” (*T*_*F*_) provides a guide for the inoculation time:


(4) $$T_{F}=T_{P}-\frac{1}{\omega }\,l\,n\,\left(\frac{V_{\phi }}{\varepsilon }\right)-\frac{1}{\mu }$$

Another feature to consider in phage therapy is that the interaction with other species influences bacterial behavior. For instance, it has been shown that the pathogenic *Escherichia coli* strain O157:H7 can adhere to substrates more easily when in the presence of *Pseudomonas aeruginosa* (14). In addition, it has been demonstrated experimentally that certain microorganisms inhibit the growth of other microbial species. For example, the commensal *Lactobacillus crispatus* slows the growth rate of the pathogens *Gardnerella viginalis* and *Neisseria gonorrhoeae* (15), whereas *Lactobacillus brevis* inhibits *Chlamydia trachomatis* (16). The opposite occurrence is also possible, with microorganisms experiencing increased growth rates when co-cultured with boosting species. For instance, the pathogens *Aggregatibacter actinomycetemcomitans* and *Candida albicans* increased the growth rate of *Streptococcus mutans*, a bacterium ubiquitous in the oral flora (17, 18). Moreover, it has been shown that phages might be able to reduce the density of a target species only in the presence of a competing microbe. For instance, phages T7 and T5 could induce the extinction *E. coli* in a culture only when *Salmonella enterica* was present (19). Microbes can, therefore, influence each other’s fitness including phagial virulence. *In vivo*, the situation is even more complicated because it is necessary to account for the immune response against both bacteria and phages (20). Within this context, the case might arise of a phage-resistant pathogen whose booster species is instead sensible to phage infection. In that case, targeting the booster species might reduce the virulence of the pathogen and hereby help the clearance of the infection.

Both Eqs. 3 and 4 were defined to account for these biological characteristics to improve the effectiveness of phage-derived antibacterial treatments. However, the issue with the definitions of *V*_*F*_ and *T*_*F*_ is that Eqs. 3 and 4 are *a posteriori* approximations which depend on the sought-after unknown quantities *T*_ϕ_ and *V*_ϕ_ required for effective therapy. Since both Eqs. 3 and 4 are non-linear equations, resolving this dependency requires solution of a system of non-linear inequalities, which is cumbersome and, without further conditions, not unique.

The aim of the present work was to use a numerical approach to identify *T*_*F*_, *V_*F*_, T_ϕ_*, and *V*_ϕ_. A decision tree algorithm was developed to explore the different outcomes of microbial consortia undergoing phage treatment and to identify the best pairs of *V*_ϕ_ and *T*_ϕ_ for achieving either active or passive treatment. The identification of a *V_ϕ_/T_ϕ_* pair will facilitate the microbiologist’s work in implementing an effective therapy. The algorithm was tested on a series of microbial consortia: (*i*) the scenario described by Payne and Jansen in their study on phage therapy; (*ii* and *iii*) dual bacteria combinations; (*iv*) two species boosting each other’s fitness.

## Materials and methods

### Microbial growth models

The focus of the present analysis was on what can be described as “allochthonous invasion,” based on the definition of *autochthonous* species (a permanent component of a specific micro-environment) and *allochthonous* (introduced anew into such a niche) species (21). At the beginning of the simulation (*t*_0_), the initial density of autochthonous species was considered higher than that of the allochthonous species, but the latter outgrew the former at a later time *t*.

Bacterial growth was implemented using logistic functions and the phage expansion was linked to the bacterial host by the following ordinary differential equations (ODEs):


(5) $$\text{Ẋ}=\mu \,X\,\left(1-\frac{N}{\kappa }\right)-\delta \,X\,P-\omega \,X-H\,\left(t\right)\,X$$

(6) İ=δ⁢X⁢P-η⁢I-ω⁢I-H⁢(t)⁢I

(7) $$\text{Ṙ}=\xi \,R\,\left(1-\frac{N}{\kappa }\right)-\omega \,R-H\,\left(t\right)\,R$$

(8) Ṗ=η⁢β⁢I-δ⁢X⁢P-λ⁢P-ω⁢P-h⁢(t)⁢P

*X* and *I* indicate the population of susceptible and infected bacteria, respectively, whereas *R* is the population of bacteria resistant to phage (*P*) infection, that is a competitive species. The terms μ and ξ indicated the growth rate of the susceptible/infected and resistant bacteria. The logistic terms were expressed as the ratio of the total bacterial population *N* to the carrying capacity κ. The phagial life-history traits were: β, burst size; δ, adsorption rate; and λ, decay rate (22). In addition, η represented the reciprocal of the latency time τ. An additional parameter ω was included for a possible wash-out of microbes; this was set to 0.15 ml/h in all models. The terms *H(t)* and *h(t)* represent the immune response against bacteria and phages, respectively. These terms were dependent on time because the immune response is not immediate (12). Since the present study focused on *in vitro* applications of phages, both *H(t)* and *h(t)* were set to zero. A list of the parameters used in the present study is reported in Table 1.

**TABLE 1:** Variables and parameters used in the present study.

Parameter	Symbol	Units	Case 1	Case 2	Case 3	Case 4
Growth rate targeted species	μ	h^–1^	0.500	0.79	0.32	0.23–0.49
Growth rate competitor species	ξ	h^–1^	–	0.22	0.20	0.18
Growth rate booster species	ν	h^–1^	–	–	–	0.24–0.42
Carrying capacity	κ	CFU × ml^–1^	6.5 × 10^6^	6.5 × 10^6^	5.0 × 10^9^	5.0 × 10^9^
Adsorption rate	δ	ml × min^–1^	1.66 × 10^–9^	5.0 × 10^–10^	5.0 × 10^–10^	4.5 × 10^–10^
Decay rate	λ	PFU × h^–1^	5.000	0.068	0.068	0.072
Burst size	β	PFU	100	150	150	115
Latency time	*t*	min	–	23	23	42
Reciprocal of latency time	η	h^–1^	5.0	2.61	2.61	1.4
Wash out rate	ω	ml × h^–1^	0.15	0.15	0.15	0.15
Simulation time	*t*	h	20	60	67	100

The examples used in the present work were derived either from batch (closed vessel) or continuous (chemostat) culture. In the former case, the growth was converted from an explicit consumption of a limiting nutritive resource to implicit consumption under the assumption that the limiting resource would have remained constant. In particular, the specific growth rates were calculated from the maximum growth rates using the Monod term:


(9) $$\mu =\frac{\mu_{m\,a\,x}\,S}{K_{S}+S}$$

with *S* being the concentration of the limiting nutrient, and *K*_*S*_ being the half-saturation constant (23, 24).

### Estimation of growth rates

The microbes’ life traits were based on information retrieved from the literature. When not provided by the experimental settings of the studies considered herein, the growth rates were calculated as a function of the bacterial population at time *t*_0_ (*N*_0_) and at time *t* (*N*_*t*_) with the formula (24):


(10) $$\mu =\frac{l\,o\,g_{10}\,\left(N_{t}\right)-l\,o\,g_{10}\,\left(N_{0}\right)}{l\,o\,g_{10}\,\left(2\right)\,\left(t-t_{0}\right)}$$

The growth rate was numerically computed as the slope of a linear model based on the bacterial densities displaying a linear distribution.

Since the model for case 4 included occurrences where the growth of a given microbe was influenced by that of another species, we addressed the use of dynamic growth rates, modifying the ODE system as follows. The growth rate of a microbe *X* cultivated alone was indicated with μ_ε_ (from the Greek ἐρημία: erēmíā, *loneliness*), whereas μ_*o*_ (from the Greek ὁμαρτῆ: homarte, *at the same time and place*) indicated its growth rate in presence of another microbe *Y* (booster) capable of enhancing the bacterial growth. Similar to *X*, *Y*’s growth rates could be indicated by *ν_ε_* and *ν_*o*_*. A consortium of a bacterium and a booster required μ terms that could shift between μ_ε_ and μ_*o*_. Since the species in the model started mixed together, the baseline growth rate was μ_*o*_, but a loneliness term ε was added to shift μ_*o*_ toward μ_ε_ with decreasing amounts of the booster species. The loneliness term was defined as: ε = Δϑ, with Δ = (μ_*o*_ – μ_ε_). The ϑ was a “quorum term” obtained by adapting the Hill function (25):


(11) $$\vartheta =\frac{\rho^{n}}{\rho^{n}+\varrho^{n}}$$

with ρ being the density of the affected species, ϱ the density of the booster species, and *n* = 1. The property of ε was that it ranged between Δ in absence of booster species (*ϑ = 1)* and Δ/*2* when the bacterial densities were equal (*ϑ = ½*). Thus, the constant growth rate μ in Eq. 5 was substituted by a function *M* defined as:


(12) $$\text{M}=f\,\left(\mu_{\varepsilon },\mu_{o},\rho ,\varrho \right)=\mu_{o}-\varepsilon =\mu_{o}-\Delta \,\vartheta =\mu_{o}$$

$$-\left(\mu_{o}-\mu_{\varepsilon }\right)\,\vartheta =\mu_{o}-\left(\mu_{o}-\mu_{\varepsilon }\right)\,\frac{\rho }{\rho +\varrho }$$

obtaining *Ẋ = f(μ_ε_, μ_*o*_, ρ, ϱ)X(1 – N/κ) – δXP – ωX* (replacing Eq. 5) and the dynamic of the booster species *Y* is given by *Ẏ = f(ν_ε_, ν_*o*_, ρ, ϱ)Y(1 – N/κ) – ωY*.

### Ensemble simulations

The computation of *V*_*F*_ and *T*_*F*_ (Eqs. 3 and 4) is in general difficult due to their non-linearity. To study how *V*_ϕ_ and *T*_ϕ_ affected the treatment outcome, an ensemble simulation with 16 384 repetitions was performed. For each iteration, the viral amounts *V*_ϕ_ and administration times *T*_ϕ_ varied. The values for viral density were randomly selected between 10^2^ and 10^12^ plaque forming units (PFU/ml), with logarithmic scaling. The range for the viral amount was chosen on the assumption that, while it is possible to make virus dilutions at any desired concentration, administering less than 100 particles per milliliter would have been both impractical and ineffective. Overly concentrated viral suspensions, on the other hand, could produce virion aggregation, reducing the efficiency of the preparation. A topic review of the literature carried out for the present work showed that virtually all phage therapies administer between 10^4^ and 10^9^ PFU/ml. Thus, the range was deemed broad enough to cover virtually all phage therapy situations. The administration times were equidistant from 0 h to the end of the simulation’s time frame.

For each iteration, the trajectory of the phage was analyzed to determine the treatment’s outcome, following the classification suggested by Payne et al. (12). Host density above 10^2^ PFU/ml at the end of the simulation marked a “failed” treatment. The therapy was considered “passive” when the phage density never exceeded 105% of the initial administered amount (*V*_ϕ_). The therapy was considered “delayed” if the peak in phage density was obtained after more than 4 h and when it was at least 105% of *V*_ϕ_. The therapy was considered “active” if the phage density increased immediately over 105% of *V*_ϕ_.

### Decision tree algorithm

To compute ranges of viral load and administration times for each type of therapy, a decision tree algorithm (26, 27) was applied to the output of the ensemble simulation. The decision tree provided a partition of the set of therapy pairs which classified each pair by their expected therapy outcome and the estimated accuracy of the prediction. The resulting ranges gave a simplified representation of the regions of “active,” “delayed,” “passive,” and “failed” outcomes. The boundary of these ranges fulfilled a similar role as the critical values *V*_*F*_ and *T*_*F*_ introduced by Payne et al. (12). In comparison to Eqs. 3 and 4, the output of the decision tree did not depend on any asymptotic assumptions on the dynamics of the concentrations. The ranges provide an *a priori* approximation of *V*_*F*_ and *T*_*F*_; therefore, these values can be used as a decision criterium for suitable therapy parameters *V*_ϕ_ and *T*_ϕ_. However, they were not as general in the sense that the ranges were only valid for fixed model parameters.

### Pareto optimal therapy pair

**FIGURE 1:**
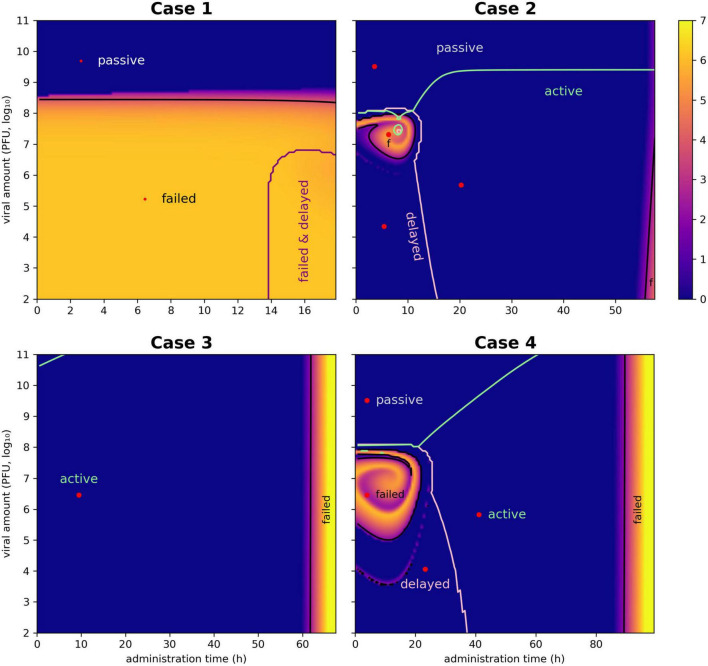
Heat maps for the selection of the most effective pair of *V*_ϕ_ and *T*_ϕ_. The ensemble simulation generates a space of viral dose and administration times whose employment lead to a different outcome. Each pixel of the plot represents the outcome of the simulation, color-coded according to the natural logarithm of the host’s density at the end of the simulation (bar on the right of the plots). There are 128 intervals in both the *x*-axis (administration time *T*_ϕ_) and the *y*-axis (viral load *V*_ϕ_), determining 16 328 simulations. The curves indicate the boundaries of the different outcomes (active, delayed, passive, and failed therapies), representing critical values equivalent to *V*_*F*_ and *T*_*F*_. The selection of optimal pairs of viral load and administration times (equivalent to *V*_ϕ_ and *T*_ϕ_) was obtained with a Pareto approach implemented with as a multi-criteria optimization problem (MCOP). These values are visualized by red dots.

The decision tree-driven classification was not sufficient to select optimal therapy pairs for a specific treatment. For example, therapy pairs at the boundary of the computed ranges are very sensitive to perturbations, resulting in undesirable outcomes for the final user. Thus, the present study solved a multi-criteria optimization problem (MCOP) (28) to provide the user with a pair of phage load (*V*_ϕ_) and administration time (*V*_ϕ_) that always resulted in the chosen outcome (“active,” “delayed,” “passive,” and “failed”). MCOP is widely used to guide the decision of treatment parameters (29). The criteria employed to achieve an effective therapy was a maximal insensitivity to perturbations combined with the shortest possible administration time. For a given therapeutic pair (*V_ϕ_, T_ϕ_*), the measure of insensitivity was the largest radius *R* of an ellipse such that all perturbed pairs *Ṽ_ϕ_, T̃_ϕ_* which satisfied the inequality (*T*_ϕ_ – *T̃_ϕ_*)^2^ + *w*_ϕ_^2^(log(*V*_ϕ_) – log(*Ṽ_ϕ_*))^2^ < *R*^2^ also yielded the desired therapy outcome (Figure 1). The scaling constant *w*_ϕ_ determined the shape of the ellipse of perturbations. For all cases in this article, the value *w_ϕ_ = 2* was used. The data from the ensemble simulation provided a fast way to approximate *R(V_ϕ_, T_ϕ_)*. The weighted sum method (28) in conjuncture with the particle swarm method (30) was used to compute Pareto optimal solutions. The approach used was prototypical in the sense that, depending on the specific application, other criteria could be chosen instead.

### Implementation

Computations were carried out in *Julia* 1.7 (31) and implemented with the packages: *DifferentialEquations* (solution of differential equations) (32); *LsqFit, Dierckx*, and *Roots* (regression); *DecisionTrees* (classification); *Optim* (optimization) (30); and *PyPlot* (plotting). Data estimation from the original plots was obtained using *WebPlotDigitizer* 4.5.^1^ Bacterial growth rates were computed using a custom function *growthRate*, built-in *R* 4.1, that selected the points of bacterial density over time most describing a continuous line and then generated a linear model on those points. The slope of the model was used as the growth rate value. Retrieval of phages species for a given bacterium was obtained by inquiring the *Virus-Host Database* during the year 2021 (33).

## Results

In the following sections, the ensemble simulations were performed for selected cases describing allochthonous invasions. The decision trees defined the limits for each type of phage-derived antibacterial treatment (“passive,” “delayed,” “active,” or “failed”), providing values equivalent to *T*_*F*_ and *V*_*F*_ (Table 2). Moreover, a pair of viral load and administration time, equivalent to the parameters *V*_ϕ_ and *T*_ϕ_, was determined by a multi-criteria optimization problem to provide the user with convenient values for implementing the chosen treatment. The cases reported below represented *in vitro* applications of phages to eliminate a target bacterium; thus, the cases did not involve the immune system. Moreover, the cases were based on the application of lytic phages; the presence of prophages in the host bacteria was not considered. The antibiotic resistance capability of the hosts and their potential virulence factors were also excluded from the modeling.

**TABLE 2:** Summary of the phage therapy outcomes obtained by decision tree approach for the cases presented in the present study.

Case	Microbial consortium	Phage	Outcome (efficacy)	*V*_ϕ_ range^†^ (PFU × ml^–1^)	*T*_ϕ_ range^‡^ (h)
1	Hypothetical*	Hypothetical	Passive (100%)	≥2.6 × 10^8^	≥0
2	Escherichia coli* + Pseudomonas aeruginosa	T4	Active (99.7%)	≤2.6 × 10^9^	15.5–56
			Delayed (100%)	≤3.7 × 10^6^	≤13.26
			Passive (100%)	≥2.6 × 10^9^	≥0
3	Escherichia coli* + Azotobacter vinelandii	T4	Active (100%)	≤4.1 × 10^10^	≤61.3
4	Streptococcus mutans* + Candida albicans + Lactobacillus reuteri	λ	Active (100%)	≤2.2 × 10^9^	36.8–89.5
			Delayed (98.7%)	≤1.2 × 10^5^	≤30.6
			Passive (100%)	≥9.6 × 10^8^	≤33

*Targeted bacterial species.

^†^The upper end of the range is 10^11^ PFU/ml.

^‡^The upper end of the range is the end of the simulation’s time.

### Case 1: Hypothetical bacterium and phage

**FIGURE 2:**
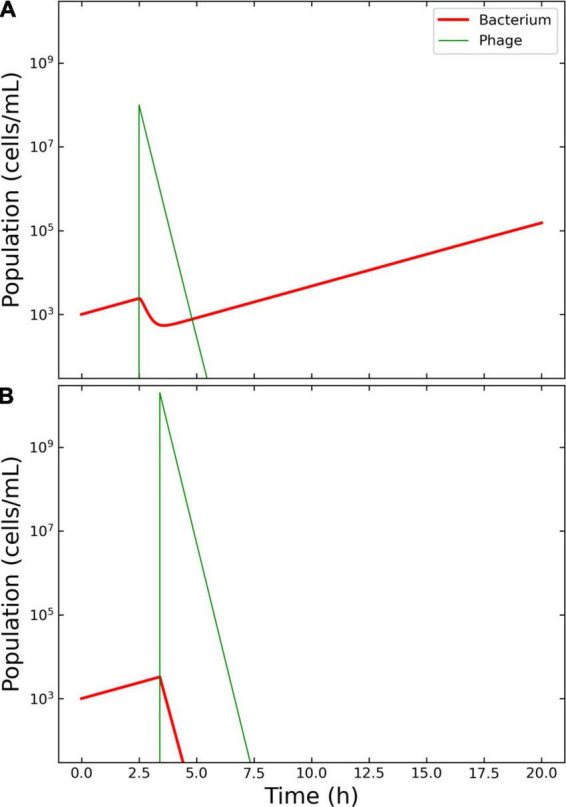
Model of the competition between hypothetical bacteria and phages. Outcome for case 1. **(A)** Failed therapy. The simulation shows a passive therapy, since there is no amplification of the phages, where the virions are depleted from the system before the bacterium could be wiped out. To note the decrease in bacterial concentration after the application of *V*_ϕ_ = 10^8^ phages at *T*_ϕ_ = 2.5 h and the increase in density of the escaped bacteria. **(B)** Effective therapy. The only effective therapy possible was passive therapy, with ample margins of administration. The Pareto-derived pair for passive therapy was: 4.5 × 10^9^ PFU/ml and 2.8 h.

Payne and Jansen described the growth of a hypothetical bacterium and the administration of its phage, highlighting four main treatment outcomes: “failed,” “passive,” “active,” and “delayed” (11). In the present study, the failed outcome was used as a base to implement an effective passive therapy. The parameters of the simulation, derived from the Payne and Jansen’s study, were as follows. Initial concentration of bacteria (*X*_0_): 1,000 colonies forming units per milliliter (CFU/ml); *V*_ϕ_: 10^8^ plaque forming units per milliliter (PFU/ml); *T*_ϕ_: 2.5 h; μ: 0.5 h^–1^; δ: 1.66 × 10^–9^ ml/min; η: 5 h^–1^; β: 100 PFU; λ: 5 PFU/h. The bacterial growth was adapted to account for a logistic growth with κ = 6.5 × 10^6^ CFU/ml and ω = 0.15 ml/h. The simulation time-frame was 20 h (Figure 2A).

The decision tree algorithm developed herein reported only one effective outcome: passive. The Pareto optimal pair of viral load and administration time was identified as 4.5 × 10^9^ PFU/ml and 2.8 h. The Pareto optimal pair of viral load and administration time was identified as 1.99 × 10^10^ and 3.4 h. The results of the therapy clearly illustrated the characteristics of an effective passive approach: there was no increase in phage density with respect to the initial input and there were no bacteria left in the environment at the end of the simulation, indicating that the infection had been cleared as required (Figure 2B).

The outcome of the therapy was dependent on the time scale of the application. While a range of 20 h allowed only for passive therapy, a longer scale (for instance, 48 h) provided also active and delayed outcomes which reduced the host below 10^2^ PFU/ml (Supplementary Figure 2). A dynamic plot was implemented to actively explore the role of the different parameters in modeling phage therapy (Supplementary File 1). The figure shows that the outcome of the phagial administration is strongly dependent on the parameters used in the computation, highlighting the fact that phage therapy is case-specific.

Remarkably, an oscillation in population density was serendipitously obtained with *V*_ϕ_ = 1.6 × 10^5^ PFU/ml and *T*_ϕ_ = 15.9 h. The model showed a first wave of phage expansion followed by bacterial decrease and a second wave of phage expansion that caused the collapse of the host population (Supplementary Figure 3A).

### Case 2: *Escherichia coli* vs. *Pseudomonas aeruginosa*

**FIGURE 3:**
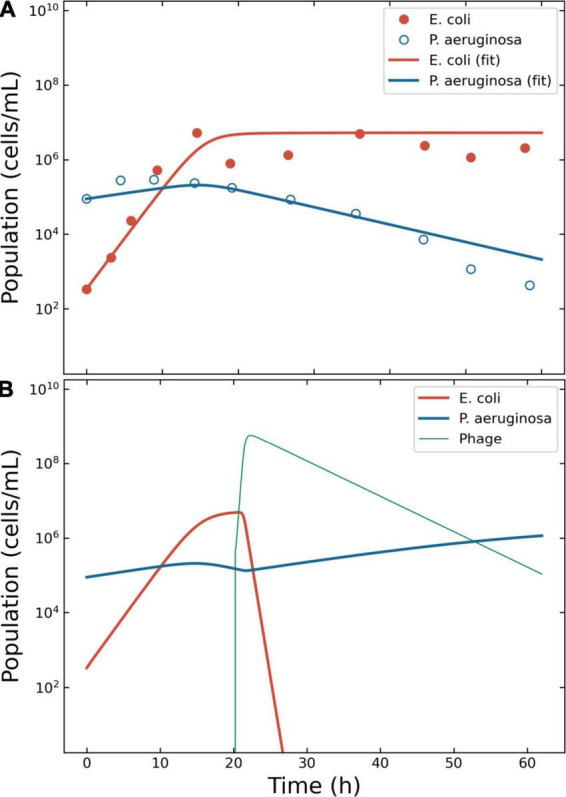
Model of the competition between *Escherichia coli* and *Pseudomonas aeruginosa*. Outcome for case 2. **(A)** Bacterial competition in absence of phages. The data estimated from the original plots for *E. coli* and *P. aeruginosa* is represented together with the fitting obtained using ODE models for *E. coli* and *P. aeruginosa*. **(B)** Bacterial competition in presence of phages. The Pareto-derived pair for active therapy was: 2.0 × 10^5^ PFU/ml and 17.7 h, leading to the extinction of the invading bacterium *E. coli* and the recovery of the resident species *P. aeruginosa*.

The growth of *Escherichia coli* C-8 and *P. aeruginosa* PAO283 was described by Hansen and Hubbell in 1980 using batch cultures (34). The life-history traits reported by this study for these bacteria were as follows. *E. coli*: yield (*Y*) 2.5 × 10^10^ cells per gram (cell/g) of limiting substance; half saturation constant (*K*_*S*_) 3.0 × 10^–6^ grams per liter (g/L) of limiting substance; μ_*max*_ = 0.81 h^–1^. *P. aeruginosa*: *Y* = 3.8 × 10^10^ cell/g; *K*_*S*_ = 3.0 × 10^–6^ g/L; *ξ_*max*_* = 0.91 h^–1^. The bacteria were growth in 100 ml flasks containing minimal medium with tryptophan as limiting nutrient, provided at an initial concentration of 1.0 × 10^–4^ g/L. The growth rates were calculated according to Eq. 9: μ = 0.79 h^–1^for *E. coli* and ξ = 0.22 h^–1^ for *P. aeruginosa*. The carrying capacity κ was estimated from the original graph at 6.5 × 10^6^ cells/ml. The initial seed of bacteria was extracted from the original graphs: *E. coli*, 334 cells/ml; *P. aeruginosa*, 88 516 cells/ml. These quantities gave a *P. aeruginosa/E. coli* ratio of 265.4, in line with the reported 200:1 for the initial densities of these bacteria. *Escherichia coli* outgrew *P. aeruginosa* about 9.2 h after the beginning of the experiment and the latter was wiped out in about 60 h. *X*_*P*_ was calculated to 10 556 cells and *T*_*P*_ at 4.4 h after the beginning of the experiment (Figure 3A).

To simulate the phage therapy, the life-history traits of the coliphage T4 were retrieved from the literature (35): δ = 5.0 × 10^–10^ ml/min; τ = 23 min (resulting in η = 2.61 h^–1^); λ = 0.068 PFU/h; β = 150 PFU. The simulation time-frame was 60 h with ω = 0.15 ml/h and κ = 6.5 × 10^6^ CFU/ml. The decision tree identified three possible effective outcomes: “passive,” “active,” and “delayed active.” The best pair of viral load and administration time for active therapy were identified in 4.8 × 10^5^ PFU/ml and 20.2 h (Figure 3B). The best pair of viral load and administration time for delayed treatment were identified in 2.2 × 10^4^ PFU/ml and 5.4 h (data not shown). The best pair of viral load and administration time for passive treatment were identified in 3.2 × 10^9^ PFU/ml and 3.6 h (data not shown).

As for case 1, an oscillation in population density was serendipitously obtained with *V*_ϕ_ = 1.0 × 10^6^ PFU/ml and *T*_ϕ_ = 10.0 h. The model showed a first wave of phage expansion followed by bacterial decrease and a second wave of phage expansion that caused the collapse of the host population (Supplementary Figure 3B).

### Case 3: *Escherichia coli* vs. *Azotobacter vinelandii*

**FIGURE 4:**
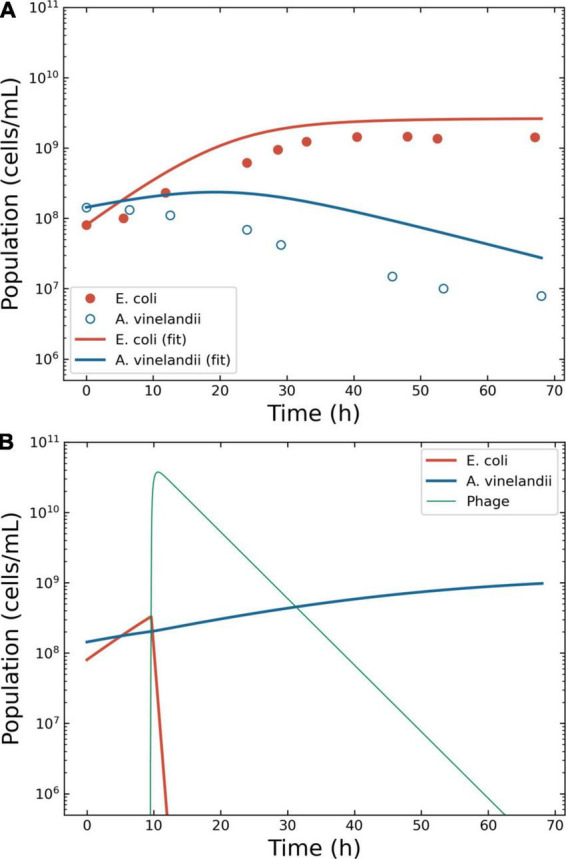
Model of the competition between *Escherichia coli* and *Azotobacter vinelandii*. Outcome for case 3. **(A)** Bacterial competition in absence of phages. The dots represent the data estimated from the original plots for *E. coli* and *A. vinelandii*, the lines the conversion to a logistic model. **(B)** Bacterial competition in presence of phages. The Pareto-derived pair for active therapy was: 9.0 × 10^9^ PFU/ml and 66.8 h, leading to the extinction of the invading bacterium *E. coli* and the recovery of the resident species *A. vinelandii*.

The growth of the bacteria *E. coli* B/r and *A. vinelandii* OP was described by Jost and collaborators in 1973 using continuous culture (36). The authors reported specific growth rates of 0.32 and 0.23 h^–1^ for *E. coli* and *A*. *vinelandii*, with *K*_*S*_ of 1.0 × 10^–7^ and 1.2 × 10^–2^, respectively. The concentration of glucose in the reactor was 0.005 mg/ml, providing maximum growth rates of 0.32 and 0.07 h^–1^ for *E. coli* and *A*. *vinelandii*. The carrying capacity κ was estimated from the original graph at 5.0 × 10^9^ CFU/ml. The calculated growth rate of *A*. *vinelandii* matched what reported in the public domain (37) but did not allow the building of a fitting model (Supplementary Figure 4). A value of ξ = 0.20 ± 0.01 was reported in the literature (38) and allowed for a better description of the data (Figure 4A). The data for the simulation were extracted from the original figure of Jost et al., providing *X*_0_ of 80 251 179 CFU/ml for *E. coli* and 143 462 884 CFU/ml for *A. vinelandii*.

The phage therapy was assumed to use coliphage T4; thus, the life traits were the same as in case 2. The simulation time-frame was 67 h with ω = 0.15 ml/h. The decision tree identified two effective therapeutic outcomes: “passive” and “active.” The Pareto optimal pair of viral load and administration time for active therapy were identified in 2.9 × 10^6^ PFU/ml and 9.5 h (Figure 4B). The Pareto optimal pair of viral load and administration time for passive therapy were identified in 1.6 × 10^6^ PFU/ml and 8.4 h (data not shown).

### Case 4: *Candida albicans*, *Streptococcus mutans*, and *Lactobacillus reuteri*

The present case investigated the effect of phage therapy on mutually synergic microbial species. *C. albicans* is an opportunistic fungus that can cause infections in multiple organs and associated to increased risk of oncogenesis (39, 40). In particular, the presence of several virulence factors allows this fungus to invade and thrive in several tissues and it can develop biofilms that protect it from antibiotic treatments (41). Being a protist, *C. albicans* is immune to phagial infection. However, experimental evidence reported that this pathogen’s growth rate is increased by booster bacteria, namely *S. mutans* (17). Consequently, targeting the booster species will provide, in theory, a “mediated phage therapy” that could reduce the pathogen’s virulence. As a proof-of-concept, we defined a hypothetical microbial consortium composed by *C. albicans* as the phage-resistant pathogen, *S. mutans* as the boosting species susceptible to phage infection, and *L. reuteri* as the commensal bacterium.

**FIGURE 5:**
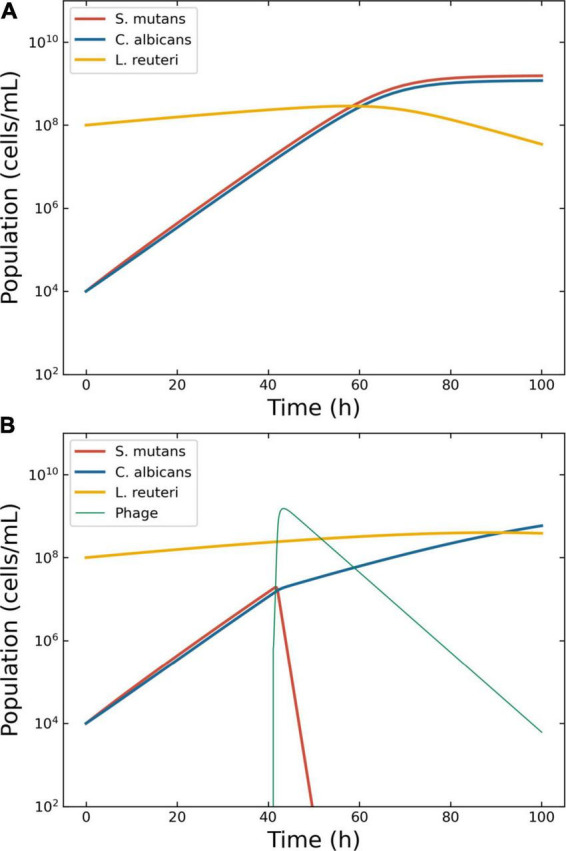
Model of the competition between *Streptococcus mutans, Candida albicans* and *Lactobacillus reuteri*. Outcome for case 4. **(A)** Bacterial competition in absence of phages. Models generated for a hypothetical consortium of two bacteria (*S. mutans* and *L. reuteri*) and one protist (*C. albicans*). The boosting species *S. mutans* (solid line) and the pathogen *C. albicans* increase each other growth rate causing a depletion in the commensal *L. reuteri*. **(B)** Bacterial competition in presence of phages. The Pareto-derived pair for active therapy was: 1.0 × 10^7^ PFU/ml and 42.6 h, leading to the extinction of the boosting bacterium *S. mutans* and consequently causing a reduction in the density of the pathogen *C. albicans* and the recovery of the commensal *L. reuteri*.

The details of the simulation were as follows. Even if not a bacterium, the growth of *C. albicans* has been modeled using logistic models (42). Thus, Eqs. 5–8 were deemed suitable to model the growth of this fungus. The growth rates of *C. albicans* and *S. mutans* were estimated from the original figures (17, 18) (Supplementary Figure 5). The density of *S. mutans* in the initial phases of growth in the presence of *C. albicans* was 8.4 ± 6.2 × 10^7^ CFU/ml; conversely, the mean density of *C. albicans* in the presence of *S. mutans* was 1.9 ± 1.1 × 10^6^ CFU/ml. Thus, the ratio *S. mutans*/*C. albicans* was 44.6. However, these measurements were taken from two different series of experiments, making it difficult to determine an accurate value of μ_*o*_ for a single consortium. The growth rate of *S. mutans* was computed at 0.23 h^–1^ when cultivated alone, and at 0.49 h^–1^ when cultivated together with *C. albicans*. Conversely, the growth rate of *C. albicans* was computed at 0.24 h^–1^ when alone and 0.42 h^–1^ when in presence of *S. mutans*. The *L. reuteri* growth rate was derived from the public domain: 0.18 h^–1^ (43) and was considered constant. The model considered an initial seed of 1 × 10^4^ CFU/ml for both *S. mutans* and *C. albicans*, and 1 × 10^8^ CFU/ml for *L. reuteri*. The model showed that both *S. mutans* and *C. albicans* grew with similar dynamics and overgrew *L. reuteri* within 60 h after the beginning of the simulation (Figure 5A). Specifically, at the end of the simulation, *C. albicans* and *L. reuteri* had densities of 1.2 × 10^9^ and 3.5 × 10^7^ CFU/ml, respectively.

The Virus-Host Database reported three phages for *S. mutans*: Streptococcus phage φAPCM01, M102, and M102AD. These phages, all belonging to the family *Siphoviridae*, were highly genetically related: M102 and M102AD shared about 91% similarity at the nucleotide level (44), and φAPCM01 shared 85% nucleotide identity with them (6). Apart for the M102AD’s adsorption rate (δ = 1.5 × 10^–10^ min^–1^ (44)), no other life traits were available in the public domain. Hence, the parameters for the present simulation were derived from another member of the *Siphoviridae* family: phage λ (35). Thus, δ = 4.5 × 10^–10^ ml/min; τ = 42 min; η = 1.4 h^–1^; λ = 0.072 PFU/h; β = 115 PFU. The carrying capacity κ was set at 5.0 × 10^9^ CFU/ml; ω = 0.15 ml/h^–1^; the simulation time-frame was 100 h.

The decision tree identified two possible therapeutic outcomes: “passive” and “active”. The Pareto optimal pair of viral load and administration time for active therapy was identified in 6.7 × 10^5^ PFU/ml and 41.0 h (Figure 5B). The best pair of viral load and administration time for passive therapy was identified in 3.2 × 10^9^ PFU/ml and 3.9 h (data not shown).

As for cases 1 and 2, an oscillation in population density was serendipitously obtained with *V*_ϕ_ = 2.9 × 10^6^ PFU/ml and *T*_ϕ_ = 3.9 h (Supplementary Figure 3C). The model showed a first wave of phage expansion followed by bacterial decrease and a second wave of phage expansion that caused the collapse of the host population.

## Discussion

In the present study, a machine learning approach was implemented to quickly analyze the possible outcomes of phage-derived antimicrobial treatments and provide the user with a pair of viral load and administration time that can result in effective antibacterial interventions. These values, equivalent to the parameters *V*_ϕ_ and *T*_ϕ_ introduced by Payne et al. (12), were extracted from a mathematical space (administration time vs. viral load) that accounted for different types of treatment (“active,” “delayed,” “passive,” and “failed”). The boundaries between these regions were equivalent to the parameters *V*_*F*_ and *T*_*F*_ defined by Payne et al. (12). The *in silico* applications presented herein (cases 1–4) did not include the immune response in the model because represented *in vitro* applications.

Nonetheless, more and more studies are reporting the role of the immune system in the effectiveness of phage therapy due to what has been called “immunephage synergy” (45–47). Notably, immunity was excluded, albeit considered, in the work by Payne and Jansen (11), Payne et al. (12). Such an assumption can be accepted considering the treatment fast enough to be completed before an immune response to both the bacterial pathogen and the phages could be instantiated. The first clinical applications of phagotherapy reported bacterial clearance as extremely rapid (48). For instance, in 1919, three young brothers were admitted to the Hôpital des Enfants-Malades, Paris, with acute dysentery. Félix d’Herelle, the first to use phages in clinical settings administered phages to them children. The children recovered in 24 h. However, contemporary clinical applications of phages last for at least 1 week (49, 50); thus, the immune response becomes a critical aspect of the therapy. Nonetheless, the immune response to phages varies among treated people. Antiphage activity of sera (AAS) was observed in about half of the patients after the tenth day of oral administration of phages (51). AAS may even be present in patients before phage therapy is initiated: phage administration resulted in a 37% increase in the baseline response in phage-naive patients; 23% of patients undergoing phagotherapy showed AAS; and it has been reported that about 80% of healthy people carry anti-phage antibodies (51, 52).

Because of the current worldwide spread of multi-drug resistant bacteria, the use of phages to clear bacterial infections is experiencing a resurgence of interest in Western countries (53). Nonetheless, to be effective, the application of phages as antibacterials should consider several factors aside from the immune response, including the host replication and rate of phage decay (12). The development of bacteria resistant to specific viral infection is also a fundamental feature to consider to obtain an effective phage treatment (54). Several models account for the bacterial development of resistance to phagial infection (55, 56). However, in the absence of experimental data, including this feature would have increased the model’s complexity without providing any real benefit to the present study. However, the increasing application of phages for eco-restoration (57), food safety (58, 59), and sterilization of surfaces (60–64) implies that phage-derived antibacterial treatments need to work even in the absence of a complementary immune response.

The present study aimed to help microbiologists involved in the medical field choosing the right amount of phages and the most effective administration time to clear an infection. While it may be tempting to administer as many phages as possible as soon as possible, Payne and Jansen’s research highlighted that doing so does not ensures the treatment’s effectiveness. Moreover, applying very high amounts of phages would trigger passive therapy, nullifying the dynamic feature that bacterial viruses have over antibiotics. The model we have introduced herein was intended to provide microbiologists involved in ecological studies with a means to assess the interactions between bacteria and phages quickly.

Case 1 was based on the aforementioned work by Payne et al. (12). The authors described (a) a failed therapy with the combination *V*_ϕ_ = 10^8^ PFU/ml, *T*_ϕ_ = 2.5 h, and (b) an effective passive therapy with *V*_ϕ_ = 1.0 × 10^9^ PFU/ml, *T*_ϕ_ = 2.5 h. The results obtained herein confirmed that, within a time frame of 20 h, only passive therapy could effectively clear the infection, and the obtained margins included the values used by Payne and Jansen to achieve effective passive therapy.

In case 2, all types of therapeutic outcome were possible. The present paper focused on active therapies, and even in this case the target bacterium (*E. coli*) was eradicated from the simulation within the allotted time. Nonetheless, the heath maps described a zone at low dispensation time (below 15 h) and intermediate viral load (around 10^7^ PFU/ml) where the treatment produced a failed outcome (Figure 1). Such a result highlighted the need to assess the outcomes of the treatment to improve its effectiveness.

Case 3 confirmed that the outcome of phage therapy is dependent on the peculiar condition of the microbial consortium. In this case, only active and passive therapy were possible. The target bacterium (*E. coli*) was removed from the *in silico* environment allowing the recovery of *A. vinelandii* as required. The analysis of this consortium was unambiguous. However, the lack of empirical data precluded the selection of the most fitting model for the growth of the bacteria. While we chose, for simplicity, the logistic growth model (Eqs. 5–8) to describe the replication of naïve bacteria, the shape of the data extracted from published experiments (36) suggested that other functions providing more sigmoid profiles, such as Holling type IV, might be viable alternatives. The precise definition of the underlying growth function was deemed of little value in the absence of experimental data.

Case 4 introduced the concept of “indirect phage therapy,” that is the targeting of a booster bacterium to decrease the pathogenesis of a phage-resistant microbe, in this case *C. albicans*. Given the morbidity and mortality associated with this fungus, driven primarily by its capacity to generate biofilms that can be colonized by a variety of microbes that facilitate horizontal gene transfer (65), methods to eradicate this opportunistic pathogen would be clinically beneficial. Since the growth rate of the microbes in the simulation was not constant but was related to the density of the partner species, we defined a dynamic growth rate for the interacting species. In the literature, there is a paucity of cases of mutually interactive microbes and their growth models. We retrieved a growth rate as a function of bacterial density in the theoretical description of cross feeding (66). Such a model required a term *c*_*x*_ to avoid the problem of infinity when the selected species’s density was equal to zero.


(13) $$\text{Ẋ}=X\,\left(\mu +b_{x\,y}\,\frac{Y}{X+c_{x}}\right)\,\left(1-\frac{X+Y}{\kappa }\right)$$

In Eq. 13, *b*_*xy*_ indicates the benefit of the species *Y* over the growth of *X*, but *c*_*x*_ does not represent a biological capacity. The function we introduced to adjust the growth rate according to the bacterial densities (Eq. 12) avoided division by zero by adapting the Hill function *aX^n^*(*X^n^* + *Y^n^*)^–1^, with *a* = 1 and *n* = 1, to the microbial densities, dispensing the need for a *c*_*x*_ term. Even in case 4, as in cases 1 and 2, there was a zone a failure at low administration times and intermediate viral loads.

Within the present framework, as in Payne et al.’s study (12), the effectiveness of the therapy was based on the complete removal of the target bacterium and assumed the absence of an immune response. While such an assumption is feasible for *in silico* systems like those included in the present study, recent models indicated that actual phage therapy, in combination or not with antibiotics, would fail without a complementary immune response (20, 67). Thus, *in vivo*, the complete removal of the target species is probably not essential to achieve remission from infection. Effective therapy will consist of phage-driven reductions in the density of the host below a threshold where the immune system can wipe out the target. Recent research has shown that phage administration activates the innate immune response and reduces harmful pro-inflammatory pathways (10, 68), but their role in the treatment outcome is still unknown. To date, the role of immunity in phage therapy remains under active investigation.

It has been shown *in vitro* that microbial competition can act synergistically with phages to reduce the density of *E. coli* (69). The *P. aeruginosa* PAO1 density decreased significantly more upon phage administration in the presence of additional species (*Staphylococcus aureus* and *S. macrophilia*, either independently or in combination) than in the absence of competitors (70). As a result, there may be a parallel between the role of the immune response *in vivo* and microbial competition *in vitro*. In both cases, phagial infection may not be enough to eliminate a specific bacterium from the environment. However, the increased selective pressure imposed by viral infection may cause a shift in microbial competition against the targeted bacterium. The role of competition in phage therapy could have significant implications for treatments that do not involve the immune response, such as in environmental applications. These data suggest a scenario where phages alone are not sufficient to eradicate a targeted bacterial host from a given micro-environment. Conversely, the simultaneous action of phages and other features (immune response, microbial competition, or antibiotics) assure the elimination of the targeted bacteria.

Moreover, the development of host-side resistance to infection has significant implications for the phage-derived antibacterial treatment. Phage-resistant mutants will counteract the phage treatment, allowing the targeted species to survive in the micro-environment (55, 71). In the present work, as in the mentioned study of Payne and Jansen, mutation was not accounted for. More refined modeling will require to include such a feature. In the absence of experimental data, the inclusion of mutation would have increased the complexity of the model without any real gain.

Consequently, the adaptation of *in silico* models to *in vivo* contexts is not trivial due to the still poorly understood additional factors involved in phage therapy. Thus, a successful *in silico* treatment does not assure the success of *in vitro* or *in vivo* implementations (72). Nonetheless, modeling is an essential part of the experimental investigation because models allow to predict results, provide explanation of empirical data, and streamline wet lab experiments (73). The method presented in the present study was devised toward microbial modeling to improve the efficacy of phage therapy by making it simple to determine the phage load and administration time ranges to be tested in the experimental settings. Our model should be regarded as a preliminary framework that can be expanded to include additional features to improve its ability to fit experimental data. For example, the current model considered the “sur-mesure” approach to phage treatment (50). In other words, a single specific phage is administered after careful characterization of a chosen pathogen. In many real-world applications, however, the most common phage therapy approach is the so-called “pret-a-porter,” where a cocktail of different phages is administered simultaneously. To account for such a phagial variability, the model would have to increase the number of phage instances to accommodate multiple life-history traits, as previously proposed (71, 74, 75). The resulting model would be much more complex than the one presented herein but, in the absence of experimental data, it would not provide any additional benefit. Similarly, the model did not consider the presence of integrated lysogenic phages in the hosts that might become activated upon infection with lytic phages to avoid unneeded complexity.

We observed indications for oscillations in population density. The peaks in host density preceded that of the phages, in accordance with the Lotka-Volterra model, namely a peak in prey density occurring before the decline in host density (76). Recent data highlighted that, in some instances of phage therapy, an invading bacterium can coexist with the resident flora, resulting in a new equilibrium (77). It is known that bacteria and phages can establish an equilibrium in the presence of specific life traits and densities (56). It has been shown that oscillatory conditions between phages and bacteria might occur when the infection rate η is within a range whose lower end (η_*c*_) is defined as:


(14) $$\eta_{c}=\frac{\omega (\delta K+\omega )}{\delta K(\beta -1)-\omega }$$

where *K* = κ(1 – ωμ^–1^) (56). The value for η_*c*_ could be calculated in 0.036, 0.001, and 0.001 h^–1^ for cases 2, 3, and 4, respectively. These critical thresholds were indeed below the values of 2.6 and 1.4 h^–1^ used in the models for cases 2–4, respectively. There is, therefore, a real risk that non-optimal viral loads might determine not the eradication of the targeted bacterium but the establishment of an unforeseen new microbial environment. The equilibrium between the target bacterium (namely a pathogen) and the dispensed lytic phage might stabilize a harmful species at low density, which might subsequently expand when the right conditions present themselves. While, *in vivo*, such species can be considered a “pathobiont” (78), at the environmental level they can still cause damage, for instance spoiling milk during cheese production. The present work will help toward the avoidance of such occurrences and increase the effectiveness of phage therapy.

The present study had some limitations. The results presented herein were only theoretical and will require empirical validation. In particular, the precision of Eq. 12 could not be determined. In the absence of experimental data, such an effort would be of very little gain; thus, the present study must be considered a proof-of-concept for further analysis. Similarly, the role of the immune response in the outcome of the treatment could not be implemented. It can be expected that expanding models modeling the interaction between bacteria and phage to include the immune response will be challenging because AAS varies depending on administration method, formulation (monotherapy vs. phage cocktails), and recipient immune status. Another major limitation of the present study was the paucity of growth rates and life-history traits. In particular, the literature on the experimental use of phages to eradicate bacteria rarely reports the exact growth rates and life-history traits of the microbes used in the experiments. The current study had to rely on a variety of information sources, which could have resulted in a distortions in the computation. Because the current model is an *in silico* approach, it is critical to empirically improve the description of bacterial and viral interactions to provide more and more accurate parameters that can increase the model’s accuracy. The increasing use of phages as antibacterial agents will necessitate a greater availability of the pool of life-history traits available to researchers and practitioners, a goal that can only be achieved through a multi-center effort. There were also relevant computational limitations. The use of a decision tree algorithm provided a tool to compute ranges for each therapy. One trade-off was that the decision tree has to approximate the domains for each therapy by rectangles. If these domains are curved the algorithm provides multiple smaller ranges to approximate the behavior around the curves. Other machine learning tools such as state vector machines are more suitable in such scenarios, but their output does not provide ranges but more complicated representations. Another computational limitation was that for the ensemble simulation the ODE system needs to be solved for many different therapy pairs. This made it challenging to configure the ODE solver optimal, since too higher tolerances lead to instability issues and but solving for all therapy pairs with low tolerances leads to very time consuming computations. Depending on the model such instability issues can cause a major problem.

In conclusion, the present study applied machine learning, in the form of a decision tree algorithm, to determine ranges for the phagial dose and administration times needed to achieve passive, active, or delayed antibacterial treatment. A multi-criteria optimization problem provided Pareto optimal treatment parameters. The procedure used herein simplified the workflow to achieve effective phage therapy. The present study also introduced the concept of mediated phage therapy, where targeting a booster bacteria might decrease the virulence of a pathogen immune to phagial infection.

## Data availability statement

The raw data supporting the conclusions of this article will be made available by the authors, without undue reservation.

## Author contributions

SP and LM conceived the ideas, designed methodology, and analyzed the data. LM collected the data and led the writing of the manuscript. SV supervised the project and granted the funds. All authors contributed critically to the drafts and gave final approval for publication.

## Footnotes

1
https://automeris.io/WebPlotDigitizer/

## References

[1] Hatfull GF. Actinobacteriophages: genomics, dynamics, and applications. *Annu Rev Virol.* (2020) 7:37–61. 10.1146/annurev-virology-122019-070009 32991269 PMC8010332

[2] Hatfull GF Dedrick RM Schooley RT. Phage therapy for antibiotic-resistant bacterial infections. *Annu Rev Med.* (2022) 73:197–211. 10.1146/annurev-med-080219-122208 34428079

[3] Brives C Pourraz J. Phage therapy as a potential solution in the fight against AMR: obstacles and possible futures. *Palgr Commun.* (2020) 6:100. 10.1057/s41599-020-0478-4

[4] Aslam S Lampley E Wooten D Karris M Benson C Strathdee S Lessons learned from the first 10 consecutive cases of intravenous bacteriophage therapy to treat multidrug-resistant bacterial infections at a single center in the United States. *Open Forum Infect Dis.* (2020) 7:ofaa389. 10.1093/ofid/ofaa389 33005701 PMC7519779

[5] Terwilliger A Clark J Karris M Hernandez-Santos H Green S Aslam S Phage therapy related microbial succession associated with successful clinical outcome for a recurrent urinary tract infection. *Viruses.* (2021) 13:2049. 10.3390/v13102049 34696479 PMC8541385

[6] Dalmasso M de Haas E Neve H Strain R Cousin FJ Stockdale SR Isolation of a novel phage with activity against *Streptococcus mutans* biofilms. *PLoS One.* (2015) 10:e0138651. 10.1371/journal.pone.0138651 26398909 PMC4580409

[7] Khalifa L Brosh Y Gelman D Coppenhagen-Glazer S Beyth S Poradosu-Cohen R Targeting Enterococcus faecalis biofilms with phage therapy. *Appl Environ Microbiol.* (2015) 81:2696–705. 10.1128/AEM.00096-15 25662974 PMC4375334

[8] Harada LK Silva EC Campos WF Del Fiol FS Vila M Dąbrowska K Biotechnological applications of bacteriophages: state of the art. *Microbiol Res.* (2018) 212–213:38–58. 10.1016/j.micres.2018.04.007 29853167

[9] Petrovic Fabijan A Khalid A Maddocks S Ho J Gilbey T Sandaradura I Phage therapy for severe bacterial infections: a narrative review. *Med J Aust.* (2020) 212:279–85. 10.5694/mja2.50355 31587298 PMC9545287

[10] Petrovic Fabijan A Lin RCY Ho J Maddocks S Ben Zakour NL Iredell JR. Safety of bacteriophage therapy in severe *Staphylococcus aureus* infection. *Nat Microbiol.* (2020) 5:465–72. 10.1038/s41564-019-0634-z 32066959

[11] Payne RJ Jansen VA. Understanding bacteriophage therapy as a density-dependent kinetic process. *J Theor Biol.* (2001) 208:37–48. 10.1006/jtbi.2000.2198 11162051

[12] Payne RJ Phil D Jansen VA. Phage therapy: the peculiar kinetics of self-replicating pharmaceuticals. *Clin Pharmacol Ther.* (2000) 68:225–30. 10.1067/mcp.2000.109520 11014403

[13] Payne RJH Jansen VAA. Pharmacokinetic principles of bacteriophage therapy. *Clin Pharmacokinet.* (2003) 42:315–25. 10.2165/00003088-200342040-00002 12648024

[14] Klayman BJ Volden PA Stewart PS Camper AK. *Escherichia coli* O157:H7 requires colonizing partner to adhere and persist in a capillary flow cell. *Environ Sci Technol.* (2009) 43:2105–11. 19368221 10.1021/es802218q

[15] Breshears LM Edwards VL Ravel J Peterson ML. *Lactobacillus crispatus* inhibits growth of *Gardnerella vaginalis* and *Neisseria gonorrhoeae* on a porcine vaginal mucosa model. *BMC Microbiol.* (2015) 15:276. 10.1186/s12866-015-0608-0 26652855 PMC4675025

[16] Mastromarino P Di Pietro M Schiavoni G Nardis C Gentile M Sessa R. Effects of vaginal lactobacilli in Chlamydia trachomatis infection. *Int J Med Microbiol.* (2014) 304:654–61. 10.1016/j.ijmm.2014.04.006 24875405

[17] Sztajer H Szafranski SP Tomasch J Reck M Nimtz M Rohde M Cross-feeding and interkingdom communication in dual-species biofilms of *Streptococcus mutans* and *Candida albicans*. *ISME J.* (2014) 8:2256–71. 10.1038/ismej.2014.73 24824668 PMC4992082

[18] Szafrański SP Deng Z-L Tomasch J Jarek M Bhuju S Rohde M Quorum sensing of *Streptococcus mutans* is activated by *Aggregatibacter actinomycetemcomitans* and by the periodontal microbiome. *BMC Genomics.* (2017) 18:238. 10.1186/s12864-017-3618-5 28320314 PMC5359896

[19] Harcombe WR Bull JJ. Impact of phages on two-species bacterial communities. *Appl Environ Microbiol.* (2005) 71:5254–9. 10.1128/AEM.71.9.5254-5259.2005 16151111 PMC1214695

[20] Leung CYJ Weitz JS. Modeling the synergistic elimination of bacteria by phage and the innate immune system. *J Theor Biol.* (2017) 429:241–52. 10.1016/j.jtbi.2017.06.037 28668337

[21] Stolp H. *Microbial ecology: Organisms, Habitats, Activities.* Cambridge, MA: Cambridge University Press (1988).

[22] García R Latz S Romero J Higuera G García K Bastías R. Bacteriophage production models: an overview. *Front Microbiol.* (2019) 10:1187. 10.3389/fmicb.2019.01187 31214139 PMC6558064

[23] Harder W Veldkamp H. Competition of marine psychrophilic bacteria at low temperatures. *Antonie Van Leeuwenhoek.* (1971) 37:51–63. 10.1007/BF02218466 5313512

[24] Prescott L Harley J Klein D. *Microbiology.* 3rd ed. Dubuque: Wm. C. Brown Publishers (1996).

[25] Hill AV. The possible effects of the aggregation of the molecules of haemoglobin on its dissociation curves. *J Physiol.* (1910) 40:i–vii. 10.1113/jphysiol.1910.sp001386

[26] Wasserman L. *All of Statistics: A Concise Course in Statistical Inference.* Berlin: Springer (2004).

[27] Hastie T Tibshirani R Friedman J. *The Elements of Statistical Learning: Data Mining, Inference, and Prediction.* Berlin: Springer (2009).

[28] Ehrgott M. *Multicriteria Optimization.* Berlin: Springer (2005).

[29] Broekhuizen H Groothuis-Oudshoorn CGM van Til JA Hummel JM IJzerman MJ. A review and classification of approaches for dealing with uncertainty in multi-criteria decision analysis for healthcare decisions. *Pharmacoeconomics.* (2015) 33:445–55. 10.1007/s40273-014-0251-x 25630758 PMC4544539

[30] Zhan Z-H Zhang J Li Y Chung HS-H. Adaptive particle swarm optimization. *IEEE Trans Syst Man Cybern Part B.* (2009) 39:1362–81. 10.1109/TSMCB.2009.2015956 19362911

[31] Perkel JM. Julia: come for the syntax, stay for the speed. *Nature.* (2019) 572:141–2. 10.1038/d41586-019-02310-3 31363196

[32] Rackauckas C Nie Q. Differentialequations.jl–a performant and feature-rich ecosystem for solving differential equations in Julia. *J Open Res Softw.* (2017) 5:15.

[33] Mihara T Nishimura Y Shimizu Y Nishiyama H Yoshikawa G Uehara H Linking virus genomes with host taxonomy. *Viruses.* (2016) 8:66. 10.3390/v8030066 26938550 PMC4810256

[34] Hansen SR Hubbell SP. Single-nutrient microbial competition: qualitative agreement between experimental and theoretically forecast outcomes. *Science.* (1980) 207:1491–3. 10.1126/science.6767274 6767274

[35] De Paepe M Leclerc M Tinsley CR Petit M-A. Bacteriophages: an underestimated role in human and animal health? *Front Cell Infect Microbiol.* (2014) 4:39. 10.3389/fcimb.2014.00039 24734220 PMC3975094

[36] Jost JL Drake JF Fredrickson AG Tsuchiya HM. Interactions of *Tetrahymena pyriformis*, *Escherichia coli*, *Azotobacter vinelandii*, and glucose in a minimal medium. *J Bacteriol.* (1973) 113:834–40. 10.1128/jb.113.2.834-840.1973 4632323 PMC285298

[37] Tsai JC Aladegbami SL Vela GR. Phosphate-limited culture of *Azotobacter vinelandii*. *J Bacteriol.* (1979) 139:639–45. 10.1128/jb.139.2.639-645.1979 457614 PMC216913

[38] Millán C Peña C Flores C Espín G Galindo E Castillo T. Improving glucose and xylose assimilation in *Azotobacter vinelandii* by adaptive laboratory evolution. *World J Microbiol Biotechnol.* (2020) 36:46. 10.1007/s11274-020-02822-5 32140791

[39] Nobile CJ Johnson AD. *Candida albicans* biofilms and human disease. *Annu Rev Microbiol.* (2015) 69:71–92. 10.1146/annurev-micro-091014-104330 26488273 PMC4930275

[40] Engku Nasrullah Satiman EAF Ahmad H Ramzi AB Abdul Wahab R Kaderi MA Wan Harun WHA The role of *Candida albicans* candidalysin ECE1 gene in oral carcinogenesis. *J Oral Pathol Med.* (2020) 49:835–41. 10.1111/jop.13014 32170981

[41] Pereira R Dos Santos Fontenelle RO de Brito EHS de Morais SM. Biofilm of *Candida albicans*: formation, regulation and resistance. *J Appl Microbiol.* (2021) 131:11–22. 10.1111/jam.14949 33249681

[42] Brusca MI Irastorza RM Cattoni DI Ozu M Chara O. Mechanisms of interaction between *Candida albicans* and *Streptococcus mutans*: an experimental and mathematical modelling study. *Acta Odontol Scand.* (2013) 71:416–23. 10.3109/00016357.2012.690530 22625873

[43] Pancheniak E de FR Maziero MT Rodriguez-León JA Parada JL Spier MR Molecular characterisation and biomass and metabolite production of *Lactobacillus reuteri* LPB P01-001: a potential probiotic. *Braz J Microbiol.* (2012) 43:135–47. 10.1590/S1517-838220120001000015 24031812 PMC3768958

[44] Delisle AL Guo M Chalmers NI Barcak GJ Rousseau GM Moineau S. Biology and genome sequence of *Streptococcus mutans* phage M102AD. *Appl Environ Microbiol.* (2012) 78:2264–71. 10.1128/AEM.07726-11 22287009 PMC3302630

[45] Tiwari BR Kim S Rahman M Kim J. Antibacterial efficacy of lytic *Pseudomonas* bacteriophage in normal and neutropenic mice models. *J Microbiol.* (2011) 49:994–9. 10.1007/s12275-011-1512-4 22203564

[46] Roach DR Leung CY Henry M Morello E Singh D Di Santo JP Synergy between the host immune system and bacteriophage is essential for successful phage therapy against an acute respiratory pathogen. *Cell Host Microbe.* (2017) 22:38e–47.e4. 10.1016/j.chom.2017.06.018 28704651

[47] Van Belleghem JD Dąbrowska K Vaneechoutte M Barr JJ Bollyky PL. Interactions between bacteriophage, bacteria, and the mammalian immune system. *Viruses.* (2018) 11:10. 10.3390/v11010010 30585199 PMC6356784

[48] Kuchment A. *The Forgotten Cure: the Past and Present of Phage Therapy.* Berlin: Springer (2014).

[49] Onsea J Soentjens P Djebara S Merabishvili M Depypere M Sriet I Bacteriophage applications for difficult-to-treat musculoskeletal infections: development of a standardized multidisciplinary treatment protocol. *Viruses.* (2019) 11:891. 10.3390/v11100891 31548497 PMC6832313

[50] Marongiu L Burkard M Lauer UM Hoelzle LE Venturelli S. Reassessment of historical clinical trials supports the effectiveness of phage therapy. *Clin Microbiol Rev.* (2022). 10.1128/cmr.00062-22 [Epub ahead of print]. 36069758 PMC9769689

[51] Żaczek M Łusiak-Szelachowska M Jończyk-Matysiak E Weber-Dąbrowska B Międzybrodzki R Owczarek B Antibody production in response to staphylococcal MS-1 phage cocktail in patients undergoing phage therapy. *Front Microbiol.* (2016) 7:1681. 10.3389/fmicb.2016.01681 27822205 PMC5075762

[52] Dabrowska K Miernikiewicz P Piotrowicz A Hodyra K Owczarek B Lecion D Immunogenicity studies of proteins forming the T4 phage head surface. *J Virol.* (2014) 88:12551–7. 10.1128/JVI.02043-14 25142581 PMC4248953

[53] Sannathimmappa MB Nambiar V Aravindakshan R. Antibiotics at the crossroads – do we have any therapeutic alternatives to control the emergence and spread of antimicrobial resistance? *J Educ Health Promot.* (2021) 10:438. 10.4103/jehp.jehp_557_21 PMC8719572 35071644

[54] Dedrick RM Smith BE Cristinziano M Freeman KG Jacobs-Sera D Belessis Y Phage therapy of mycobacterium infections: compassionate-use of phages in twenty patients with drug-resistant mycobacterial disease. *Clin Infect Dis.* (2022) ciac453. 10.1093/cid/ciac453 35676823 PMC9825826

[55] Lenski RE Levin BR. Constraints on the coevolution of bacteria and virulent phage: a model, some experiments, and predictions for natural communities. *Am Nat.* (1985) 125:585–602. 10.1086/284364

[56] Weitz JS. *Quantitative Viral Ecology: Dynamics of Viruses and Their Microbial Hosts.* Princeton, NJ: Princeton University Press (2016).

[57] Sharma RS Karmakar S Kumar P Mishra V. Application of filamentous phages in environment: a tectonic shift in the science and practice of ecorestoration. *Ecol Evol.* (2019) 9:2263–304. 10.1002/ece3.4743 30847110 PMC6392359

[58] Olson EG Micciche AC Rothrock MJJ Yang Y Ricke SC. Application of bacteriophages to limit campylobacter in poultry production. *Front Microbiol.* (2021) 12:458721. 10.3389/fmicb.2021.458721 35069459 PMC8766974

[59] Wang Z Zhao X. The application and research progress of bacteriophages in food safety. *J Appl Microbiol.* (2022) 133:2137–47. 10.1111/jam.15555 35353432

[60] Liu Y Mi Z Niu W An X Yuan X Liu H Potential of a lytic bacteriophage to disrupt *Acinetobacter baumannii* biofilms in vitro. *Future Microbiol.* (2016) 11:1383–93. 10.2217/fmb-2016-0104 27538011

[61] D’Accolti M Soffritti I Lanzoni L Bisi M Volta A Mazzacane S Effective elimination of Staphylococcal contamination from hospital surfaces by a bacteriophage-probiotic sanitation strategy: a monocentric study. *Microb Biotechnol.* (2019) 12:742–51. 10.1111/1751-7915.13415 31025530 PMC6559196

[62] Pinto G Almeida C Azeredo J. Bacteriophages to control Shiga toxin-producing *E. coli* – safety and regulatory challenges. *Crit Rev Biotechnol.* (2020) 40:1081–97. 10.1080/07388551.2020.1805719 32811194

[63] Stachler E Kull A Julian TR. Bacteriophage treatment before chemical disinfection can enhance removal of plastic-surface-associated *Pseudomonas aeruginosa*. *Appl Environ Microbiol.* (2021) 87:e0098021. 10.1128/AEM.00980-21 34347517 PMC8478462

[64] Liao Y-T Zhang Y Salvador A Harden LA Wu VCH. Characterization of a T4-like bacteriophage vB_EcoM-Sa45lw as a potential biocontrol agent for shiga toxin-producing *Escherichia coli* O45 contaminated on mung bean seeds. *Microbiol Spectr.* (2022) 10:e0222021. 10.1128/spectrum.02220-21 35107386 PMC8809338

[65] Ponde NO Lortal L Ramage G Naglik JR Richardson JP. Candida albicans biofilms and polymicrobial interactions. *Crit Rev Microbiol.* (2021) 47:91–111. 10.1080/1040841X.2020.1843400 33482069 PMC7903066

[66] Bull JJ Harcombe WR. Population dynamics constrain the cooperative evolution of cross-feeding. *PLoS One.* (2009) 4:e4115. 10.1371/journal.pone.0004115 19127304 PMC2614108

[67] Rodriguez-Gonzalez RA Leung CY Chan BK Turner PE Weitz JS. Quantitative models of phage-antibiotic combination therapy. *mSystems.* (2020) 5:e00756-19. 10.1128/mSystems.00756-19 32019835 PMC7002117

[68] Khatami A Lin RCY Petrovic-Fabijan A Alkalay-Oren S Almuzam S Britton PN Bacterial lysis, autophagy and innate immune responses during adjunctive phage therapy in a child. *EMBO Mol Med.* (2021) 13:e13936. 10.15252/emmm.202113936 34369652 PMC8422068

[69] Laird T Abraham R Sahibzada S Abraham S O’Dea M. In Vitro demonstration of targeted phage therapy and competitive exclusion as a novel strategy for decolonization of extended-spectrum-cephalosporin-resistant *Escherichia coli*. *Appl Environ Microbiol.* (2022) 88:e0227621. 10.1128/aem.02276-21 35254097 PMC9004402

[70] Mumford R Friman V-P. Bacterial competition and quorum-sensing signalling shape the eco-evolutionary outcomes of model in vitro phage therapy. *Evol Appl.* (2017) 10:161–9. 10.1111/eva.12435 28127392 PMC5253424

[71] Bull JJ Vegge CS Schmerer M Chaudhry WN Levin BR. Phenotypic resistance and the dynamics of bacterial escape from phage control. *PLoS One.* (2014) 9:e94690. 10.1371/journal.pone.0094690 24743264 PMC3990542

[72] Melo LDR Oliveira H Pires DP Dabrowska K Azeredo J. Phage therapy efficacy: a review of the last 10 years of preclinical studies. *Crit Rev Microbiol.* (2020) 46:78–99. 10.1080/1040841X.2020.1729695 32091280

[73] Styles KM Brown AT Sagona AP. A Review of using mathematical modeling to improve our understanding of bacteriophage, bacteria, and eukaryotic interactions. *Front Microbiol.* (2021) 12:724767. 10.3389/fmicb.2021.724767 34621252 PMC8490754

[74] Cortez MH Weitz JS. Coevolution can reverse predator-prey cycles. *Proc Natl Acad Sci U.S.A.* (2014) 111:7486–91. 10.1073/pnas.1317693111 24799689 PMC4034221

[75] Chaudhry WN Pleška M Shah NN Weiss H McCall IC Meyer JR Leaky resistance and the conditions for the existence of lytic bacteriophage. *PLoS Biol.* (2018) 16:e2005971. 10.1371/journal.pbio.2005971 30114198 PMC6112682

[76] Bulmer MG. Phase relations in the ten-year cycle. *J Anim Ecol.* (1975) 44:609–21. 10.2307/3614

[77] Javaudin F Latour C Debarbieux L Lamy-Besnier Q. Intestinal bacteriophage therapy: looking for optimal efficacy. *Clin Microbiol Rev.* (2021) 34:e0013621. 10.1128/CMR.00136-21 34668734 PMC8528124

[78] Jochum L Stecher B. Label or concept – what is a pathobiont? *Trends Microbiol.* (2020) 28:789–92. 10.1016/j.tim.2020.04.011 32376073

