# Supplementary material for: Determination of phage load and administration time in simulated occurrences of antibacterial treatments
Source: Front Med (Lausanne). 2022 Oct 28;9:1040457. doi: 10.3389/fmed.2022.1040457 (PMC9650209; doi:10.3389/fmed.2022.1040457)
Supplement: Supplementary file 1 [file Data_Sheet_1.docx]

Supplementary Material

# Supplementary Data

A dynamic plot of case 1 is available at: https://observablehq.com/@3784219e03ed337e/interactive-phage-simulation.

# Supplementary Figures and Tables

## Supplementary Figures


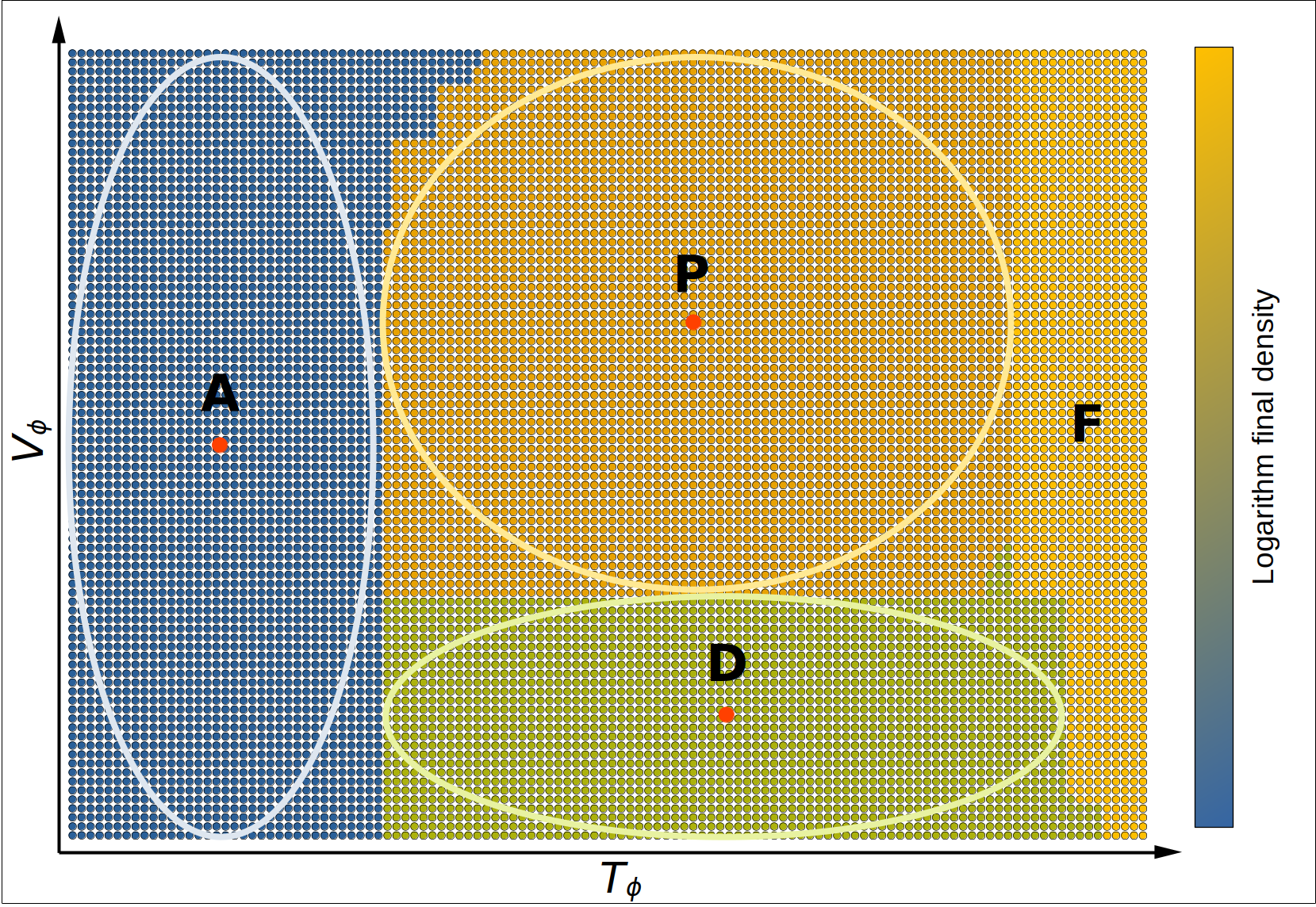
**Supplementary Figure 1. Output of the ensemble simulations.**

Exemplification of the process for the classification of the results obtained from the ensemble simulations. Each dot represents a simulation implemented using a combination of viral load (*V_ϕ_*) and administration time (*T_ϕ_*). The dot is shaded according to the resulting final density of the host. The bar on the side provides an indication of such density, with values defined as logarithm of the density expressed in CFU/mL. There are 128 values on both the *x*-axis and the *y*-axis, providing 16 384 dots that define a heat map. It is possible to discriminate regions within these maps that define the outcome of the treatment: ‘active’ (A), ‘delayed’ (D), ‘passive’ (P), and ‘failed’ (F). For the first three classes, the MCOP procure consists in fitting a circle within a specific region. The center if the circle (red dot) provided the most likely combination of *V_ϕ_* and *V_ϕ_* for the chosen type of treatment. Failed treatments do not require Pareto-derived pairs.


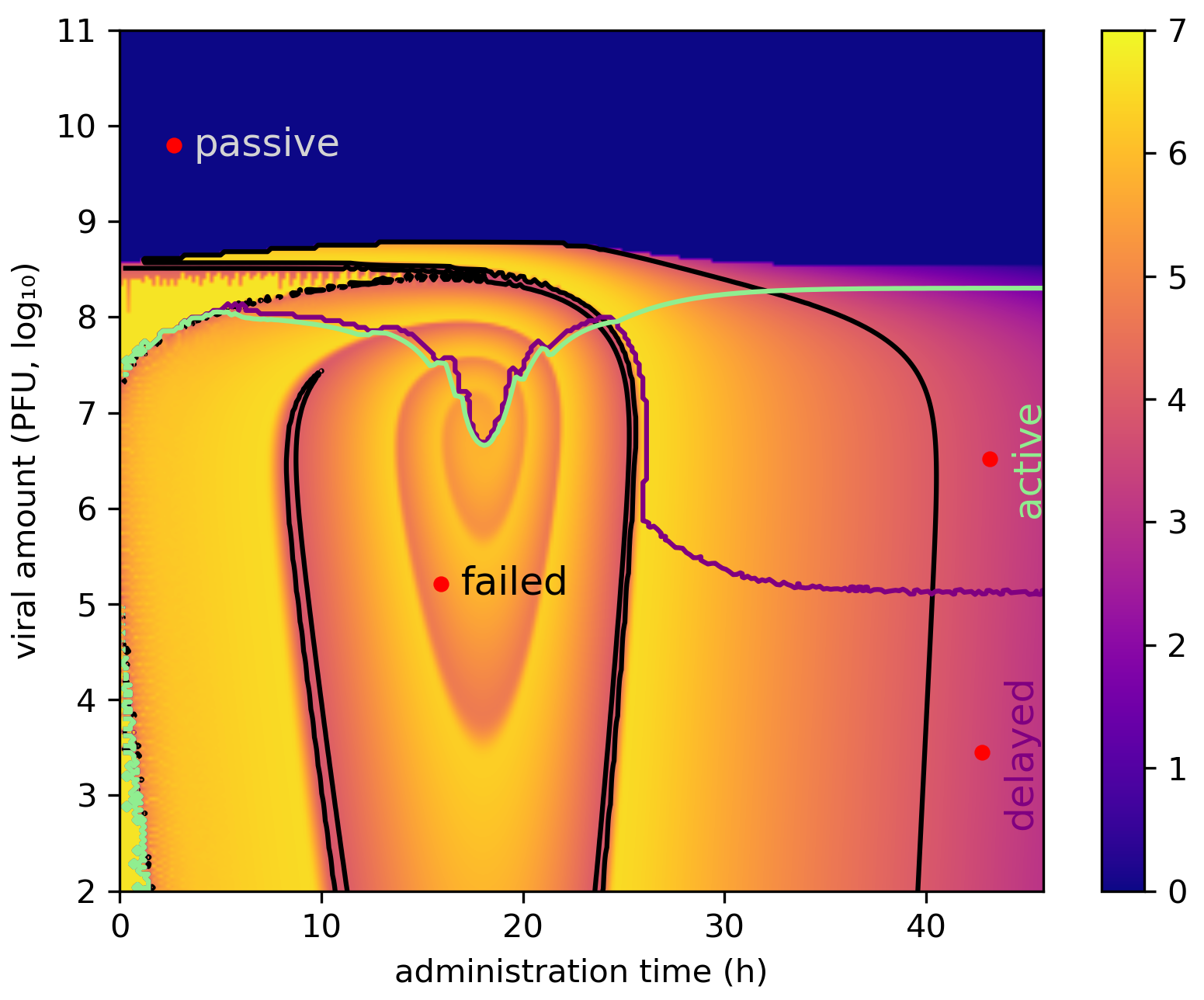
**Supplementary Figure 2. heat map for case 1 with extended simulation time.**

It was possible to obtain active and delayed outcomes in addition to passive treatment by increasing the simulation time of case 1 from 20 to 48 h. However, in contrast to the passive treatment, the active and delayed treatments in this case do only reduce the host within the given simulation time frame.


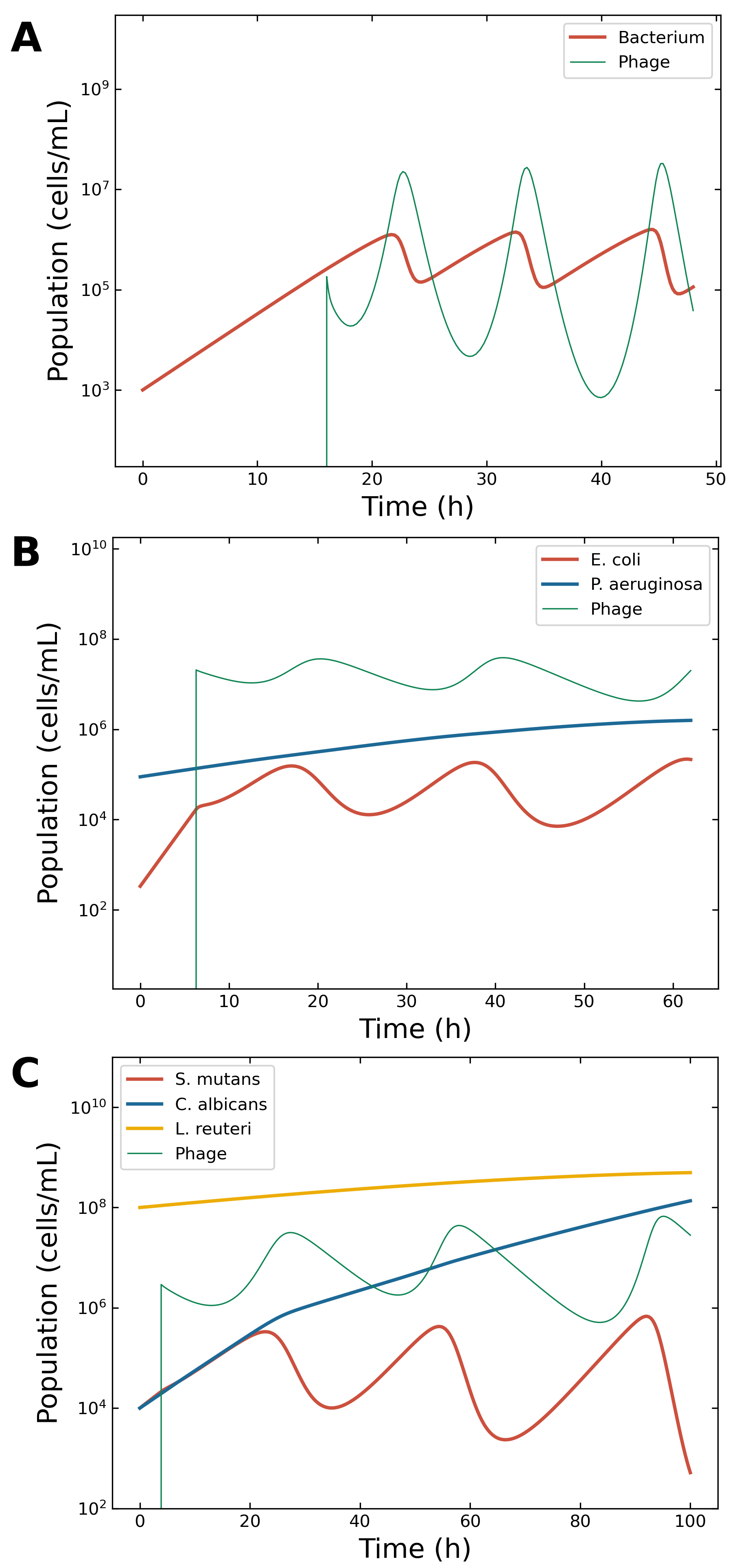


**Supplementary Figure 3. Cycling occurrences in host-prey densities.**

Oscillation in prey and predator densities for case 1 (A), case 2 (B) and case 4 (C). In certain conditions, bacteria and phages can establish a more dynamic interaction where the depletion of the host is followed by the depletion of the prey, which then results in the expansion of the host. In this case, the second wave of host expansion was followed by eradicating the bacterium resulting in effective treatment. Note how the peak in phagial density precedes the decline of bacterial density, a characteristic of the Lotka-Volterra framework.

**
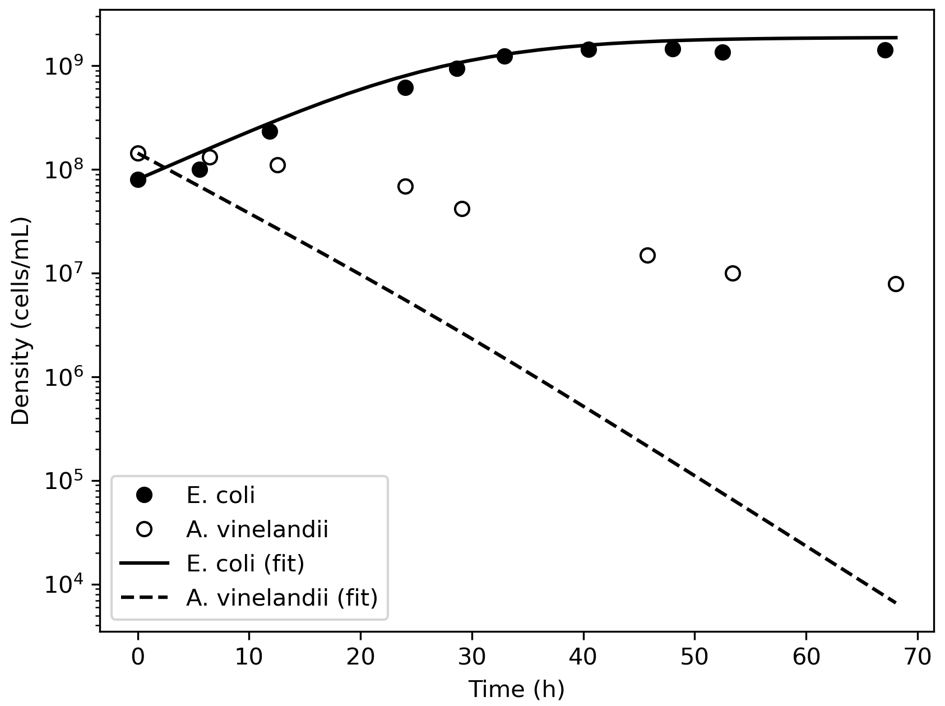
**

**Supplementary Figure 4. Model of the competition between *E. coli* and *A. vinelandii* using the original growth rates.**

Plot of the data extrapolated from the study by Jost *et al*. (1973) (●○). The logistic model obtained using the growth rates reported in the study fitted the *E. coli* (solid line) data but not that for *A. vinelandii* (dashed line).


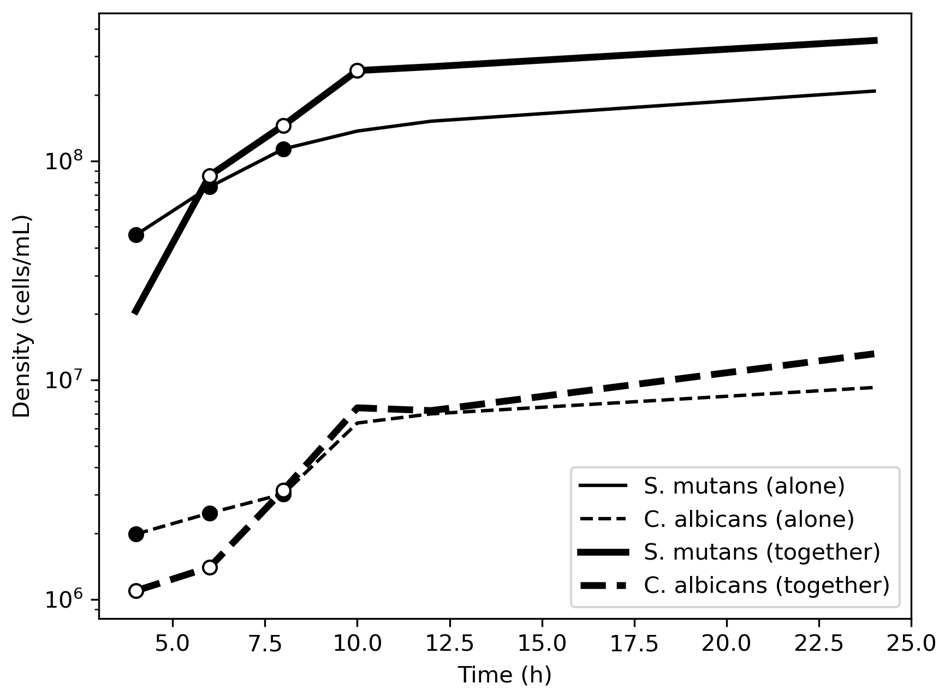


**Supplementary Figure 5. Estimation of growth rates for *Streptococcus mutans* and *Candida albicans.***

Densities over time of *S. mutans* (solid lines) and *C. albicans* (dashed lines) extracted from the work of Jost *et al.* (1973). The microbes were grown alone (thin lines) or in combination (thick lines). The data-points used to build the linear models that provided the growth rates are depicted.
